# Stillbirth Without Viability: An Intrapartum Diagnosis of Twin Reversed Arterial Perfusion (TRAP) Sequence in Monochorionic Twin Pregnancy

**DOI:** 10.7759/cureus.87555

**Published:** 2025-07-08

**Authors:** Sujata Siwatch, Rohan Sodhi, Gaurav Khastgir, Pooja Sikka

**Affiliations:** 1 Obstetrics and Gynaecology, Postgraduate Institute of Medical Education and Research, Chandigarh, IND

**Keywords:** acardiac twin, complicated twin pregnancy, monochorionic diamnionic twin, pump twin, twin gestation, twin pregnancy complications, ultrasonography in twin pregnancy

## Abstract

Twin reversed arterial perfusion (TRAP) sequence is a rare complication of monochorionic twin pregnancies, characterised by a nonviable acardiac twin perfused by a structurally normal "pump" twin. We report a case of a 33-year-old woman, G4P2012, who presented at 29 weeks in advanced labour with a history of inadequate antenatal care. She delivered a live male neonate, followed by the unexpected delivery of an intrauterine mass initially suspected to be a fibroid. Further evaluation revealed the mass to be an acardiac twin, and placental examination confirmed monochorionic diamniotic placentation, establishing the diagnosis of TRAP sequence. This case highlights the critical importance of early and detailed antenatal ultrasonography in twin pregnancies. It also raises important questions regarding the classification of stillbirths in the context of severe congenital anomalies incompatible with life.

## Introduction

Twin reversed arterial perfusion (TRAP) sequence is a rare congenital anomaly unique to monochorionic twin pregnancies, characterised by a structurally normal "pump" twin and a severely malformed "acardiac" twin that lacks a functional heart. It is estimated to occur in one in 35,000 pregnancies and represents about 1% of all monochorionic twin gestations [1]. With improved imaging and assisted reproduction, the reported incidence is increasing [2]. The acardiac twin, being nonviable, survives solely on reversed arterial perfusion from the pump twin, which is at risk of high-output cardiac failure, preterm labour, and other complications [1]. Early diagnosis is essential to optimise outcomes. We present a unique case of TRAP sequence diagnosed only after vaginal delivery of the pump twin at 29 weeks, due to inadequate antenatal care. With advances in medical facilities improving the viability of preterm babies, the gestational cut-off for defining a stillbirth is decreasing [3]. However, definitions can be confusing, as seen in our case of a twin pregnancy with an acardiac twin that has no chance of viability. Should this be labelled a stillbirth, knowing it is not viable at birth? Our case highlights the importance of thorough ultrasound examinations and vigilant monitoring in twin pregnancies. It also prompts a reconsideration of the criteria used to define stillbirths in infants with malformations incompatible with life.

## Case presentation

A 33-year-old woman, G4P2012, presented at 29 weeks in advanced labour. She had two previous vaginal deliveries at term and a spontaneous first trimester abortion. Her antenatal care was notably inadequate, limited to two self-initiated ultrasound scans. The first, performed at 11 weeks, revealed a twin gestation: one live foetus and one missed abortion. A subsequent scan at 22 weeks identified a single live foetus consistent with gestational age, along with a fundal fibroid.

**Figure 1:**
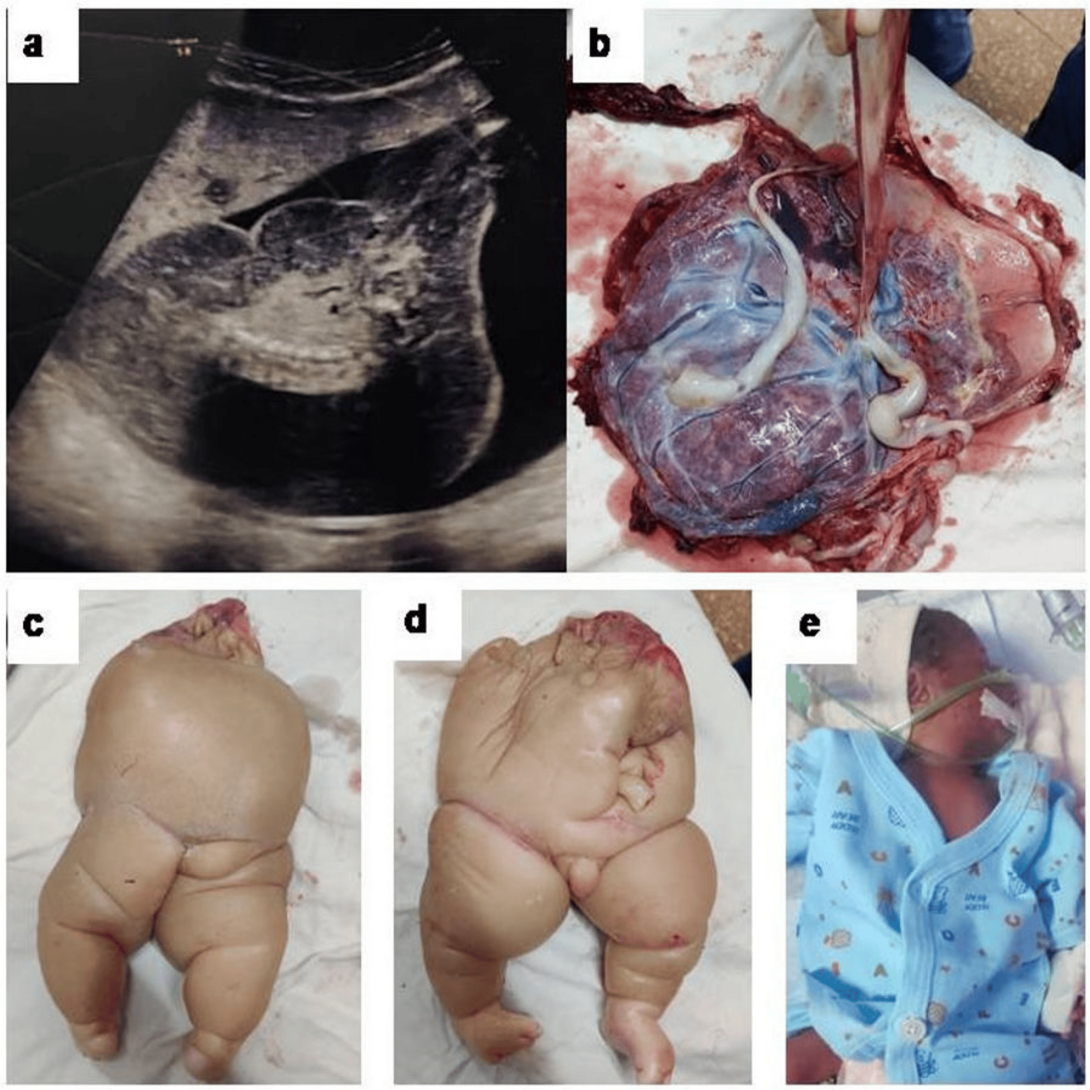
Ultrasonographic and gross images of the acardiac twin and pump twin a) Ultrasound picture of acardiac twin seen as a large heterogenous intrauterine mass with spine-like calcification b) Monochorionic diamniotic placenta c-d) Acardiac twin e) Pump twin

On presentation, her per vaginal findings revealed a fully dilated cervix with the vertex at -2 station and she soon vaginally delivered a live male neonate weighing 1147 grams (Figure 1E). Following delivery, a firm, smooth mass was palpated through the cervix. A provisional diagnosis of a fundal fibroid was considered. Upon reviewing the earlier scans, suspicion was raised for a twin gestation with an acardiac twin.

Bedside ultrasonography (USG) revealed a 15 x 10 cm hypo-heteroechoic intrauterine mass with spine-like calcifications and areas of vascularity (Figure 1A). Vaginal exploration under general anaesthesia led to the delivery of an acardiac twin weighing 1133 grams (Figures 1C-1D). Examination of the placenta confirmed monochorionic diamniotic placentation (Figure 1B), consistent with the diagnosis of TRAP sequence. The newborn was shifted to the Neonatal Intensive Care Unit (NICU) after birth, but succumbed at 30 hours of life due to prematurity-related complications. The immediate postpartum period was uneventful, and the mother was discharged on the fourth postnatal day.

## Discussion

TRAP sequence is a rare congenital anomaly characterised by the presence of a structurally normal "pump" twin and an anomalous "acardiac" twin, often lacking a functional heart and other vital organs. It usually occurs in monozygotic twins, with a frequency of one in 35,000 pregnancies and is common in female twins [1]. However, with advancements in USG, prenatal diagnosis and assisted conception, van Gemert et al. report the incidence to be rising towards 2.6% of monochorionic pregnancies [2]. Acardiac foetuses are classified as acephalus (60-75%) with absent head and upper body; anceps (~10%) with partial development including a rudimentary head; acormus (~5%) with only a head; amorphus (~20%) with no recognisable structures and myelacephalus with a partially developed head with identifiable upper limbs [4-6].

The etiopathogenesis involves abnormal placental arterio-arterial vascular communication between twins, leading to an imbalance of interfoetal circulation. [7]. Unequal embryonic splitting and genetic abnormalities leading to defective embryogenesis have also been hypothesised [8,9]. The pump twin receives high-pressure blood flow, while the perfused twin suffers from reversed deoxygenated blood flow, impairing its development. Without a functioning heart, the acardiac twin relies on the pump twin’s circulation in a parasitic way. 

Early diagnosis is crucial for deciding therapy and planning the ideal time of delivery. The characteristic ultrasound feature is the reversed blood flow into the acardiac twin via its umbilical artery, observed with colour Doppler ultrasound. Additionally, important findings include a foetus with multiple severe malformations, subcutaneous oedema, large fluid accumulations, and an absence of a heart [1]. 

Moore et al. have described a regression equation to deduce the weight (in grams) of the anomalous twin [10]. A key prognostic factor is the ratio of acardiac twin weight to donor twin weight (%). When this ratio exceeds 70%, preterm delivery occurs in 90%, polyhydramnios in 40%, and congestive heart failure in 30% [11]. However, estimating the weight of the acardiac twin is difficult. A low pulsatility index in the donor twin's umbilical artery on colour Doppler indicates a poor prognosis.

The pump twin with cardiac activity has a high risk for congestive cardiac failure. Other complications include preterm labour and premature rupture of membranes secondary to uterine overdistension. It may also suffer from hypoxia and growth restriction due to deoxygenated blood entering it via the vascular anastomotic channels. These factors contribute to a high perinatal mortality rate of around 55% for the pump twin [12]. Early diagnosis allows time for intrauterine radiofrequency ablation (RFA) and prevents complications for the viable twin [13].

Currently, there is no established consensus regarding the ideal time of delivery. Some authors have suggested adopting a conservative management approach in case the weight ratio is less than 70% and there is no evidence of heart failure in the pump twin [14]. Weekly ultrasonography is recommended in these cases. In cases with radiological evidence of heart failure, polyhydramnios or features of hydrops foetalis, an aggressive early first-trimester intervention is recommended [8]. Platt et al. were the first to suggest an interventional approach [15]. There are several approaches targeted at occluding or destroying the aberrant arteriovenous connections. These include alcohol embolisation, RFA, microwave ablation, thermocoagulation, laser coagulation and high intensity focused ultrasound [7,16,17]. However, studies have demonstrated that RFA is the most effective with a high safety index [7]. 

A stillbirth is a baby born without signs of life as indicated by the absence of breathing, heartbeat, or definite movements of voluntary muscles beyond a specified gestational age threshold. The definition of stillbirths has been evolving. With the advancement of medical facilities, as the ability to salvage preterm neonates is improving, the gestational cutoff for defining a ‘stillbirth’ is reducing [3]. However, cases like the TRAP sequence challenge these definitions. The acardiac twin, while technically born without signs of life, is nonviable by nature of its malformation. Whether such births should be recorded as stillbirths remains debatable and invites reconsideration of classification criteria for congenital anomalies incompatible with life.

## Conclusions

Our case demonstrates a TRAP sequence in monochorionic gestation, diagnosed after delivery of the first twin. Adequate antenatal care, early diagnosis and management would have avoided the complications of the trapped acardiac twin. This highlights the need for detailed USG and close monitoring in twin pregnancies. It also provokes a re-examination of the criteria for defining stillbirths in babies with malformations incompatible with life. A multidisciplinary approach involving obstetricians, radiologists and neonatologists is essential for optimising outcomes of the pump twin and for planning the appropriate time of delivery.
